# Unraveling the multifaceted resilience of arsenic resistant bacterium *Deinococcus indicus*

**DOI:** 10.3389/fmicb.2023.1240798

**Published:** 2023-08-24

**Authors:** André G. Gouveia, Bruno A. Salgueiro, Dean O. Ranmar, Wilson D. T. Antunes, Peter Kirchweger, Ofra Golani, Sharon G. Wolf, Michael Elbaum, Pedro M. Matias, Célia V. Romão

**Affiliations:** ^1^Instituto de Tecnologia Química e Biológica António Xavier (ITQB NOVA), Universidade Nova de Lisboa, Oeiras, Portugal; ^2^Department of Life Sciences Core Facilities, Weizmann Institute of Science, Rehovot, Israel; ^3^Instituto Universitário Militar, Centro de Investigação da Academia Militar (CINAMIL), Unidade Militar Laboratorial de Defesa Biológica e Química (UMLDBQ), Lisbon, Portugal; ^4^Department of Chemical and Biological Physics, Weizmann Institute of Science, Rehovot, Israel; ^5^Department of Chemical Research Support, Weizmann Institute of Science, Rehovot, Israel; ^6^Instituto de Biologia Experimental e Tecnológica (iBET), Oeiras, Portugal

**Keywords:** metals, UV-C, oxidative stress, PolyP granules, arsenate reductase, arsenate

## Abstract

Arsenic (As) is a toxic heavy metal widely found in the environment that severely undermines the integrity of water resources. Bioremediation of toxic compounds is an appellative sustainable technology with a balanced cost-effective setup. To pave the way for the potential use of *Deinococcus indicus,* an arsenic resistant bacterium, as a platform for arsenic bioremediation, an extensive characterization of its resistance to cellular insults is paramount. A comparative analysis of *D. indicus* cells grown in two rich nutrient media conditions (M53 and TGY) revealed distinct resistance patterns when cells are subjected to stress via UV-C and methyl viologen (MV). Cells grown in M53 demonstrated higher resistance to both UV-C and MV. Moreover, cells grow to higher density upon exposure to 25 mM As(V) in M53 in comparison with TGY. This analysis is pivotal for the culture of microbial species in batch culture bioreactors for bioremediation purposes. We also demonstrate for the first time the presence of polyphosphate granules in *D. indicus* which are also found in a few *Deinococcus* species. To extend our analysis, we also characterized *Di*ArsC2 (arsenate reductase) involved in arsenic detoxification and structurally determined different states, revealing the structural evidence for a catalytic cysteine triple redox system. These results contribute for our understanding into the *D. indicus* resistance mechanism against stress conditions.

## Introduction

Arsenic presents high toxicity and has been classified as carcinogenic, mutagenic and teratogenic element (Machado et al., 1999; National Research Council, 2001; Wang et al., 2017). Due to the electronic configuration of its valence orbitals, it presents a unique chemical nature that allows several changes in oxidation states and bonding configurations, forming a wide diversity of inorganic and organic species (O’Day, 2006). In nature, four oxidation states are found, namely −3 (arsine), 0 (arsenic), +3 (arsenite) and +5 (arsenate); the latter two are of the utmost interest due to their high toxicity and predominance in aquatic environments (Bissen et al., 2003; James et al., 2017). Arsenite, As(III), is mainly present in groundwater under reducing conditions while arsenate, As(V) is present in oxidizing conditions (Bissen et al., 2003; James et al., 2017). As a metalloid, arsenic is a pnictogen found in group 15 of the periodic table and presents high physicochemical similarities with phosphorus (Dani, 2011; Fekry et al., 2011; Tawfik and Viola, 2011; Varadwaj et al., 2022). The uptake of arsenate by cells follows through the phosphorus pathways mainly by phosphate transporters (Rosenberg et al., 1977; Jiang et al., 2014; Garbinski et al., 2019). Arsenate (AsO_4_^−3^) and phosphate (PO_4_^−3^) share similar chemical speciation, thus as a chemical analog of phosphorus, arsenate is able to act as substrate for several enzymes, leading to harmful cellular effects (Strawn, 2018). To cope with arsenic, most living organisms have evolved mechanisms to circumvent its toxicity. Most bacterial species bear an arsenic resistance (*ars*) operon that encompasses at least three main core genes: (1) an arsenic responsive repressor (*arsR*), (2) As(III) efflux permease (*arsB*) and (3) arsenate reductase (*arsC*) (Páez-Espino et al., 2009; Wang et al., 2016; Yang and Rosen, 2016). *Deinococcus indicus* Wt/1aT was isolated from an arsenic contaminated aquifer in West Bengal, India and it is the first known species of the *Deinococcus* genus able to withstand high concentrations of both arsenate and arsenite (Suresh et al., 2004). *D. indicus* contains the typical *arsRBC* operon that encodes two regulatory proteins (ArsR1 and ArsR2), two arsenate reductases (ArsC2 and ArsC3) and an arsenite efflux pump protein (ArsB). Besides this *ars* operon, it also encodes an additional arsenate reductase, ArsC1 (Suresh et al., 2004; Tawfik and Viola, 2011; Ranganathan et al., 2023).

The *Deinococcus* genus is widespread in the environment, colonizing extreme habitats such as deserts, hot springs and polar regions (Hirsch et al., 2004; De Groot et al., 2005; Makk et al., 2016; Jin et al., 2019). Members of this genus present high resistance to radiation and desiccation (Jin et al., 2019). Nevertheless, with the exception of *Deinococcus radiodurans*, the model organism for radiation resistance, and *Deinococcus geothermalis,* a biofilm forming organism that heavily affects the paper industry, most *Deinococcus* sp. have been poorly characterized to date (Kolari et al., 2001, 2003; Peltola et al., 2008; Slade and Radman, 2011; Gerber et al., 2015; Lim et al., 2019). *D. radiodurans* and *D. geothermalis* have shown high efficiency in the detoxification of toxic materials (Brim et al., 2000, 2003; Misra et al., 2012; Choi et al., 2017; Manobala et al., 2019). Nonetheless, before exploring the bioremediation capabilities of an organism, the characterization of its resistance to cellular insults is imperative. Moreover, the analysis of culture conditions is vital for the application of microbial species in batch bioreactors for toxic waste removal (Tekere, 2019). So far, *D. indicus* ability to survive under UV-B radiation was evaluated and no information is available regarding oxidative stress resistance (Suresh et al., 2004).

Here, *D. indicus* resistance to UV-C, oxidative stress and arsenate was investigated in two rich nutritional media (M53 and TGY). Interestingly, a media-dependent response was observed for UV-C and methyl viologen (MV) damage, with *D. indicus* presenting higher resistance in M53. Upon exposure to As(V), *D. indicus* was able to grow in the presence of 25 mM of As(V) and to tolerate 50 mM of As(V) while *D. radiodurans* had its growth compromised in the presence of 50 fold lower concentration. Polyphosphate granules have been shown to be involved in heavy metal detoxification in different microorganisms, and indeed elemental analysis by cryogenic scanning transmission electron microscopy with energy dispersive X-ray spectroscopy (STEM-EDX) revealed the presence of prominent polyphosphate granules in *D. indicus*. To extend our understanding of arsenic resistance in *D. indicus*, the crystal structure of *Di*ArsC2 arsenate reductase was determined in two states: native and bound to arsenic.

## Materials and methods

### *Deinococcus* cell growth

*Deinococcus radiodurans* R1 and *D. indicus* Wt/1aT (MTCC 4913) were grown either in: M53 medium, 1.0% (w/v) casein yeast peptone (Sigma-Aldrich), 0.5% (w/v) yeast extract (VWR Chemicals), 0.5% (w/v) glucose (Carl Roth) and 0.5% (w/v) NaCl (Merck); or Tryptone Glucose Yeast extract (TGY) medium, 1.0% (w/v) tryptone (VWR Chemicals), 0.5% (w/v) yeast extract (VWR Chemicals) and 0.1% (w/v) glucose (Carl Roth). For all assays, cells were grown at 30°C and 150 rpm agitation; for solid cultures, 1.5% (w/v) agar-agar (Carl Roth) was added. For each assay, two sequential subcultures were grown for 14–16 h prior to the final culture. Final cultures were diluted to an early-exponential phase either (Optical density at 600 nm (OD_600_) of 0.2) for arsenate exposure growth curves and STEM-EDX or (OD_600_ = 0.5) for oxidative stress, in-plate arsenate stress and UV assays.

### Oxidative and arsenate stress response

Oxidative and arsenate stress assays were performed by adapting the disk diffusion method (Bauer et al., 1966; Hudzicki, 2012). Briefly, 400 μl of cells grown in either M53 or TGY (at an OD_600_ = 2) were evenly spread on the surface of an agar plate (120.5 × 120.5 mm) using a cotton swab and allowed to dry for 15 min before the application of the disks (6 mm, PRAT DUMAS). Afterwards, oxidative stress inducer compounds or arsenic (20 μl) were applied on top of the disk in increasing concentrations. Plates were incubated for 3 days and halos were measured using Fiji/ImageJ (Schindelin et al., 2012). Oxidative stress compounds: (1) Methyl viologen dichloride, MV (C_12_H_14_Cl_2_N_2_· xH_2_O, Sigma-Aldrich); (2) Hydrogen peroxide 30% (w/w), H_2_O_2_ (Sigma-Aldrich). Arsenic stress compound: sodium arsenate dibasic heptahydrate 98%, As(V), (HAsNa_2_O_4_ · 7H_2_O, Sigma Aldrich).

### UV-C radiation survival curves

Ultraviolet (UV) irradiations were performed at UV-C (254 nm) with a fluence of 3 mW/cm^2^ throughout the assay. Fluence was assessed using a MS-100 optical radiometer with a MS 125 UVC sensor (Ultra-Violet Products, Upland, CA). *D. indicus* cells grown in either M53 and TGY (at an OD_600_ = 2) were serially diluted and plated in duplicate for each dose. Plates were air dried for 15 min and placed under the UV lamp to be exposed from 300 to 1800 J/m^2^ (in increments of 300 J/m^2^ which corresponds to an exposure of 10 s). Afterwards, plates were incubated for 3 days and the colony forming units (CFU) were assessed. The average CFU mL^−1^ of non-irradiated sample aliquots represented 100% survival. The surviving fraction at a given dose is the average CFU mL^−1^ from each irradiated sample (N) divided by the average CFU mL^−1^ of the non-irradiated sample (N_0_). Survival curves were obtained by plotting the logarithm of N/N_0_ versus the dose. To determine the survival curve parameters, the multi-hit model was applied (equation 1), S(D) = Survival fraction (where 1 is 100%); D = Dose (J/m^2^); K = inactivation constant and n = extrapolation number (obtained by the intercept of the extrapolated semi-log straight-line), (Severin et al., 1983; Kowalski et al., 2000; Kowalski, 2009). For statistical intercomparison of the different D_10_ values extrapolated from the survival curves of M53 and TGY a Student’s t-test was used and *Ρ* ≤ 0.05 were considered statistically significant (De La Vega et al., 2005).


(1) $$S\left(D\right)=1-{\left(1-e^{-kD}\right)}^{n}\;$$

### Arsenic resistance growth curves

Arsenic resistance was assessed by adding As(V) to broth cultures (OD_600_ = 0.3) for both M53 and TGY. *D. indicus* cells were incubated with 10, 25 and 50 mM while *D. radiodurans* was incubated with 0.05, 0.5, 1, 5, 10, and 20 mM of As(V). Growth curves were obtained by plotting the measured OD_600_ versus time.

### SEM of *Deinococcus indicus*

*Deinococcus indicus* cells were grown in M53 or TGY in 24 well plates for 3 days at 30°C. Grown cells were washed with Phosphate Buffer Saline (PBS) and applied over a lamella. Samples were fixed using a mixture of 2.5% glutaraldehyde and 1% formaldehyde in 0.1 M sodium cacodylate buffer for 1 h at room temperature. Afterwards, samples were washed 3 times with 0.1 M of sodium cacodylate buffer and dehydrated by sequential washing with increasing concentration of ethanol solutions (50, 70, 90, and 100%). Ethanol was removed and tert-butyl alcohol was added. Samples were incubated at 4°C for 30 min followed by 1 h in a vacuum desiccator and kept at room temperature until the scanning electron microscopy (SEM) analyses were performed. Samples were incubated for 10 min with 2% solution of osmium tetroxide and gold sputtered with 8 nm gold in an electron sputter (Cressington 108). Imaging was performed using a Hitachi TM3030Plus scanning electron microscope, at 1.5 kV.

### STEM imaging and EDX of *Deinococcus indicus*

*Deinococcus indicus* cells were grown in M53 broth media (OD_600_ of 0.3) and washed twice with M53 at 4000 rpm, 4°C. Afterwards, cells were diluted to an appropriated concentration and 4 μL deposited onto a glow-discharged Quantifoil copper grid. The grids were blotted, and plunged into liquid ethane with an automated plunger (EM-GP, Leica Microsystems). STEM imaging was performed under cryogenic conditions with a field-emission Talos F200X (S)TEM microscope (Thermo Fisher Scientific) at 200 kV accelerating voltage (extraction voltage 3,850 V, gun lens 4, spot size 9), with a condenser aperture of 70 μm in microprobe mode with a semi convergence angle of 2.1 mrad. Energy-dispersive X-ray spectroscopy (EDX) was performed using an energy dispersive spectrometer encompassing a Super-X G2 detector that features 4 silicon drift detector (SDDs) for substantially enhanced sensitivity, which is critical for trace element detection, with 8 μs dwell time per pixel, and dispersion of 5 eV per channel. Acquired images and EDX data were processed using Velox™ (Thermo Fisher Scientific) software followed by analysis in ImageJ (Schindelin et al., 2012).

### Co-localized *Deinococcus indicus*-granule size metrics

To identify *D. indicus* cells and the granules residing within them, grids were imaged using a Talos Arctica 200 kV FEG STEM (Thermo Fisher Scientific) applying the same settings described above with a few variations: spot size 4, camera length 160 mm and 6.66 nm pixel size with 1 μs dwell time per pixel. To quantify the size and distribution of granules within the bacteria, we used tiling and stitching images taken using TFS Tomography software to cover large field of view with multiple bacteria. Stitching was done using Fiji Grid/Collection stitching plugin (Preibisch et al., 2009). Segmentation of the individual bacteria and individual granules residing within them was performed and the number of granules and their average and total size within each bacterium was measured. To segment the bacteria and granules, we used a workflow combining Ilastik (Berg et al., 2019), pixel classifier followed by further processing using dedicated Fiji macro (Schindelin et al., 2012). We used multiple fields of view from different conditions for training two-stage machine-learning classifier in Ilastik “autocontext” approach to classify pixels into four categories: bacteria, granules, background and unclassified. The trained classifier was applied to the stitched images in Fiji (Schindelin et al., 2012). Bacteria were segmented based on connected component analysis of a filled mask of all pixels classified as bacteria, followed by size (1.6 < size [μm^2^] < 6) and shape filtering (circularity >0.2). Granules were then segmented based on connected component analysis of a mask of all pixels classified as granules within segmented bacteria and further filtered by size (0.012 < size [μm^2^] < 0.12) and shape (circularity >0.3). Manual correction was applied to correct the segmentation of some missed or falsely detected bacteria. The size and number of all valid bacteria and granules were extracted and analyzed.

### Sequence alignment and phylogenetic tree

Multiple sequence alignments were performed using ClustalW (Thompson et al., 1994). Aligned sequences were used as input and the phylogenetic tree was generated using maximum likelihood (ML) in IQ-TREE2 (version 2.2.2.6) (Minh et al., 2020), software with the mod LG + G4 substitution model. The model was selected by running the first tree using the model finder option (Kalyaanamoorthy et al., 2017). The final tree was generated by performing bootstrap analysis of 1,000 data sets and was treated and displayed using the Interactive Tree Of Life (iTOL) (version 6) web service (Letunic and Bork, 2021).

### Protein expression and purification

*Di*ArsC2 gene was cloned into a pET28a(+) plasmid that contains an His-tag followed by a TEV cleavage site (GenScript Inc.). The overexpression of *Di*ArsC2 was obtained in *Escherichia coli* BL21 (Gold) cells transformed with the plasmid pET28a(+)-*Di*ArsC2 and grown in Luria-Bertani (LB) medium at 37°C, 180 rpm. Cells at an OD_600_ of 0.7 were induced with 500 μM of isopropyl *β*-D-thiogalactopyranoside (NZYTech) and grown overnight at 20°C. Afterward, the cells were harvested and resuspended in lysis buffer [20 mM HEPES pH 7, 10% glycerol, 300 mM NaCl, 10 mM MgCl_2_, 1 μg/ml DNase I and 0.1 mg/ml Lysozyme (Sigma-Aldrich)]. Cells were disrupted by 5 cycles of freeze and thaw, and the soluble fraction containing overexpressed *Di*ArsC2 was obtained by centrifugation at 25,931 x *g*, 20 min at 4°C. The supernatant was loaded onto a His Trap excel column (Cytiva) using as binding buffer 20 mM HEPES pH 7, 250 mM NaCl, 10% (v/v) glycerol, and 10 mM imidazole, and the elution buffer was 20 mM HEPES pH 7, 250 mM NaCl, 10% (v/v) glycerol and 1 M imidazole. *Di*ArsC2 eluted at 300 mM of imidazole and was further loaded onto a desalting column (Hiprep^tm^ 26/10 Desalting, Cytiva) using 20 mM HEPES pH 7, 250 mM NaCl, 10% glycerol (v/v). Afterward, the *Di*ArsC2 His-tag was cleaved by adding Tobacco Etch Virus (TEV) protease (Sigma) at 12°C with gentle shaking for 14 h. *Di*ArsC2 was loaded onto His Trap excel (Cytiva) using the same buffers as described above. The final purification step was done by size exclusion chromatography (Superdex 75 10/300 GL, Cytiva), with the buffer 20 mM HEPES pH 7 and 250 mM NaCl. The protein was pure as judged by sodium dodecyl sulphate polyacrylamide gel electrophoresis analysis and concentrated to 21 mg/mL prior to being used for crystallization trials.

### Crystallization and X-ray diffraction data collection

Crystallization screens for *Di*ArsC2 were setup at the nL scale in a Crystallization Robot Mosquito LCP (SPT Labtech) with triple sitting drop 96-well plate (TTP Labtech) and using commercial screens Structure 1 and 2 (Molecular Dimensions). Needle- and plate-like crystals appeared in several conditions, three of which were selected for further optimization: #37 (0.2 M Sodium acetate trihydrate, 0.1 M Tris pH 8.5 and 30% (w/v) PEG 4000); #46 (0.05 M Potassium phosphate monobasic and 20% (w/v) PEG 8000); and #47 (15% (w/v) PEG1500). These were optimized by vapor diffusion using 2 μl hanging drops equilibrated against 500 μl reservoir solution in XRL 24-well crystallization plates (Molecular Dimensions), at 20 and 4°C with varying ratios of protein and crystallization solution in each drop. For the final optimized crystallization condition, a ratio of 1:1 (protein to crystallization solution) with 0.2 M Sodium acetate trihydrate, 0.1 M Tris pH 8.5 and 20% (w/v) PEG 4000 for *Di*ArsC2 native (henceforth referred to as *Di*ArsC2) at 4°C. In the case of *Di*ArsC2 bound to arsenic (*Di*ArsC2-As), a ratio of 1:0.8 (protein to crystallization solution) was used using 0.2 M Sodium acetate trihydrate, 0.1 M Tris pH 8.5 and 30% (w/v) PEG 4000, plus 0.2 μl of 0.1 M As(V) at 20°C. *Di*ArsC2 crystals appeared after 7 days while *Di*ArsC2-As crystals appeared after 3 days. Crystals were dipped in a cryoprotecting solution that consisted of the crystallization buffer supplemented with 10% (v/v) PEG 400 and flash-cooled in liquid nitrogen.

### Structure determination, refinement, and quality assessment

*Di*ArsC2 crystals were initially screened in-house using Cu Kα radiation with 1.5418 Å wavelength in a Bruker AXS Proteum Pt135 CCD detector system coupled to an Incoatec Microfocus X-ray Source with Montel mirrors. Bruker Proteum software package were used to process and scale the images. The structure was solved by Molecular Replacement using MORDA (Vagin and Lebedev, 2015) and using as phasing model the structure of arsenate reductase from *Bacillus subtilis* (PDB code: 1JL3) (Bennett et al., 2001). In addition *Di*ArsC2-As crystal was also tested in-house and the structure was solved by PHASER (McCoy et al., 2007) in the PHENIX suite (Adams et al., 2010; Liebschner et al., 2019) using the *Di*ArsC2 as phasing model.

Diffraction data to higher resolution were collected at the XALOC beamline BL13 of the ALBA Synchrotron (Barcelona, Spain) (Juanhuix et al., 2014). The images were processed with autoPROC (Vonrhein et al., 2011), which makes use of XDS (Kabsch, 2010) and the CCP4 suite (Potterton et al., 2003) for integration and conversion of integrated intensities to structure factors. Each dataset was integrated with XDS, followed by POINTLESS (Evans, 2011) for space-group determination, scaling and merging with AIMLESS and application of an anisotropic resolution cut-off at CC^1/2^ < 0.3 with STARANISO (Tickle et al., 2018). The crystals belonged to the monoclinic space group *P*2_1_ and contained two molecules per asymmetric unit, as estimated by the Mathews coefficient probability (Matthews, 1968; Kantardjieff and Rupp, 2003). The data processing statistics are given in Supplementary Table S1. Higher resolution crystallographic structures for both *Di*ArsC2 and *Di*ArsC2-As were determined by molecular replacement using PHASER-MR (McCoy et al., 2007) via the CCP4 Graphics User Interface (Potterton et al., 2003) or PHENIX Suite (Adams et al., 2010; Liebschner et al., 2019), and using the *Di*ArsC2-As structure obtained from the in-house data as the phasing model. For *Di*ArsC2, an initial refinement was done with REFMAC (Murshudov et al., 1997) and continued with PHENIX.REFINE (Terwilliger et al., 2007; Adams et al., 2010; Afonine et al., 2012) while for *Di*ArsC2-As refinements were done using PHENIX.REFINE. Throughout the refinement, the model was periodically checked and corrected with COOT against σ_A_-weighted 2|F_o_|-|F_c_| and |F_o_|-|F_c_| electron-density maps. Solvent molecules were added manually by inspection of electron-density maps in COOT (Emsley and Cowtan, 2004). TLS (translation-libration-screw) reciprocal space refinement was carried out for both structures. Hydrogen atoms were included in calculated positions with the PHENIX.READYSET tool and isotropic displacement parameters (ADPs) were refined for all non-hydrogen atoms. The final structures were validated using MOLPROBITY (Chen et al., 2010). Refinement statistics are given in Supplementary Table S2. Structure factors and associated structure coordinates of *Di*ArsC2 and *Di*ArsC2-As were deposited in the Protein Data bank (Berman et al., 2003) with accession code 8P6M and 8P5N, respectively (Supplementary Table S2).

A simple ensemble refinement protocol in PHENIX was run for both final structures *Di*ArsC2 and *Di*ArsC2-As considering one domain per independent molecule in the asymmetric unit, of the crystal structure, and using 80% of the non-hydrogen atoms to include in the TLS fitting of the atomic displacement parameters (ADPs) from a previous refinement with isotropic ADPs (Burnley et al., 2012). The secondary structure was determined running PROCHECK within CCP4 Graphics User Interface (Laskowski et al., 1993; Potterton et al., 2003). Figures of the structures were prepared using PyMOL (Delano, 2022).

## Results

### *Deinococcus indicus* response to oxidative stress and UV-C

**Figure 1:**
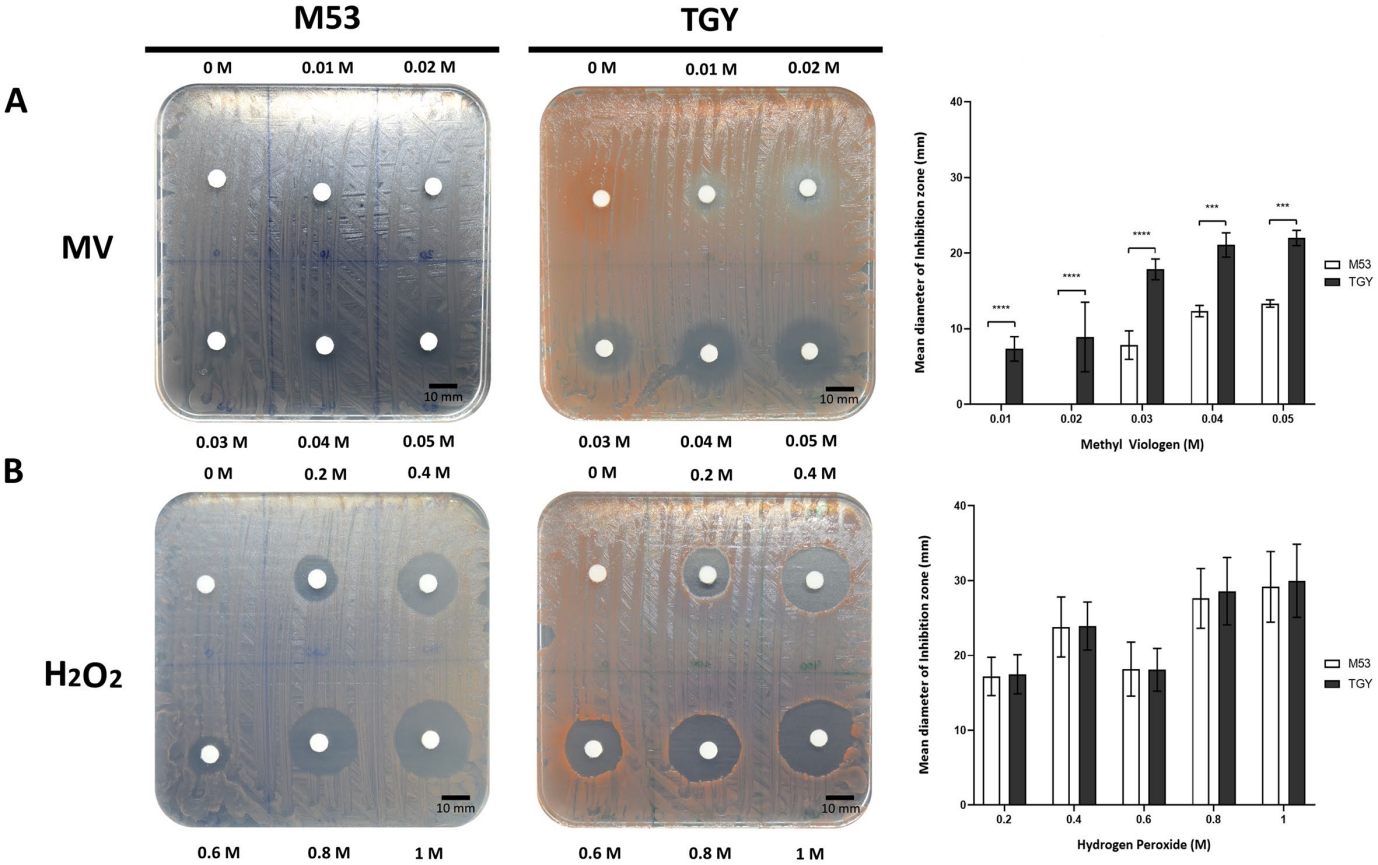
Survival of *D. indicus* at mid-exponential phase (OD_600_ = 2.0) to different oxidative stress conditions. **(A)** Cells exposed to methyl viologen (MV). **(B)** Cells exposed to hydrogen peroxide (H_2_O_2_). **(A,B)** Representative images of adapted Kirby-Bauer assays to cells grown in M53 and TGY exposed to increasing concentrations (left and middle panels). Inhibition halos correspond to the mean diameter of the inhibition zone (mm), (right panel). The error bars represent the standard deviations from three replicates of four independent experiments (*n* = 4). *p-*values were obtained by Two-Way ANOVA. *p* ≤ 0.001 and *p* ≤ 0.0001 are represented as: *** and ****, respectively.

**Figure 2:**
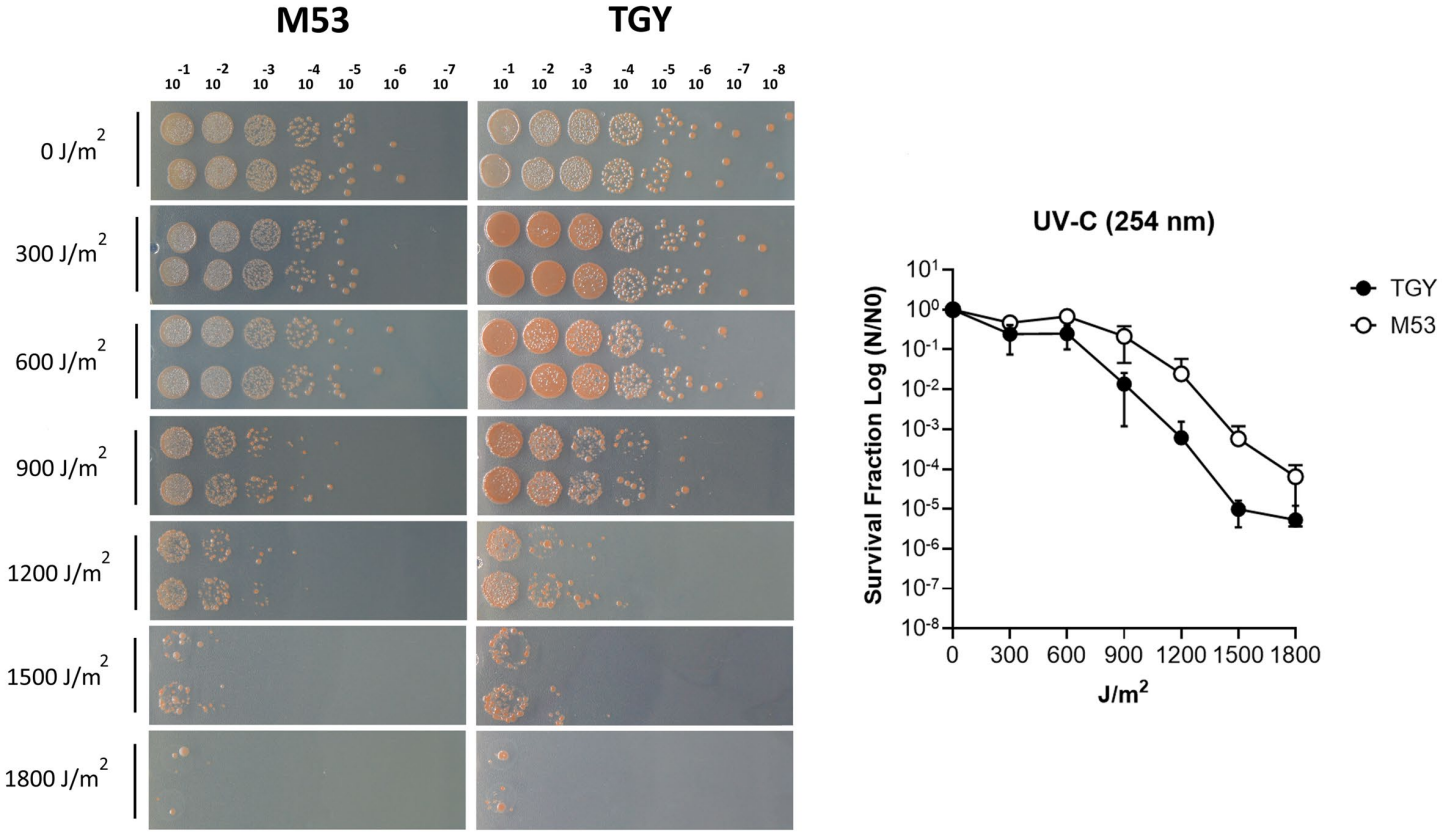
UV-C irradiation survival of *D. indicus* in M53 and TGY. **(A)** Representative image of *D. indicus* CFUs exposed to increasing doses of UV-C (254 nm) radiation in M53 and TGY**. (B)** Survival curves of *D. indicus*. The survival fraction was calculated by dividing the CFUs of UV-C irradiated cells (N) by the CFUs of non-irradiated cells (*N*_0_). Filled circles correspond to cells grown in TGY while open circles correspond to cells grown in M53. The error bars represent the standard deviation of four independent experiments (*n* = 4).

*Deinococcus* species are known for their extreme tolerance to radiation and oxidative stress (Lim et al., 2019). To evaluate *D. indicus* tolerance, we subjected the cells grown in M53 and TGY to hydrogen peroxide (H_2_O_2_) and methyl viologen (MV). MV is known to catalyze the formation of superoxide anion (O^2•-^), (Halliwell and Gutteridge, 1999; Halliwell, 2006). *D. indicus* cells presented higher resistance to H_2_O_2_ than to MV in both media (Figure 1). Upon exposure to MV, cells grown in M53 showed higher resistance than the ones grown in TGY. In fact, at 20 mM of MV, cells in M53 were neither inhibited nor had their cell growth compromised. However, the same was not observed when directly applying H_2_O_2_, in which case the cells responded in the same way independently of the culture medium (Figure 1B). *D. indicus* cells were also exposed to UV-C irradiation. It is known that when UV radiation is applied to microbial populations a slight delay is often observed before the onset of an exponential decay (Cerf, 1977; Munakata et al., 1991; Pruitt and Kamau, 1993; Kowalski et al., 2000; Kowalski, 2009). This effect is usually named as a shoulder curve due to its shape and for this reason the multi-hit model was applied to determine curve parameters and extrapolate the D_10_ (i.e., the dose required to yield 10% survival) for the cells grown in both culture media (Severin et al., 1983; Kowalski et al., 2000; Kowalski, 2009). Interestingly, in M53 cells exhibited significant higher resistance to UV-C in contrast to the ones grown in TGY (Figure 2). This was verified by calculating the D_10_ values (*Ρ* ≤ 0.05): in M53 a dose of 809 ± 66 J/m^2^ (30s exposure) is required to eliminate 90% of cells, while in TGY 619 ± 82 J/m^2^ (20s exposure) was sufficient to achieve the same result.

### Arsenate tolerance assays

**Figure 3:**
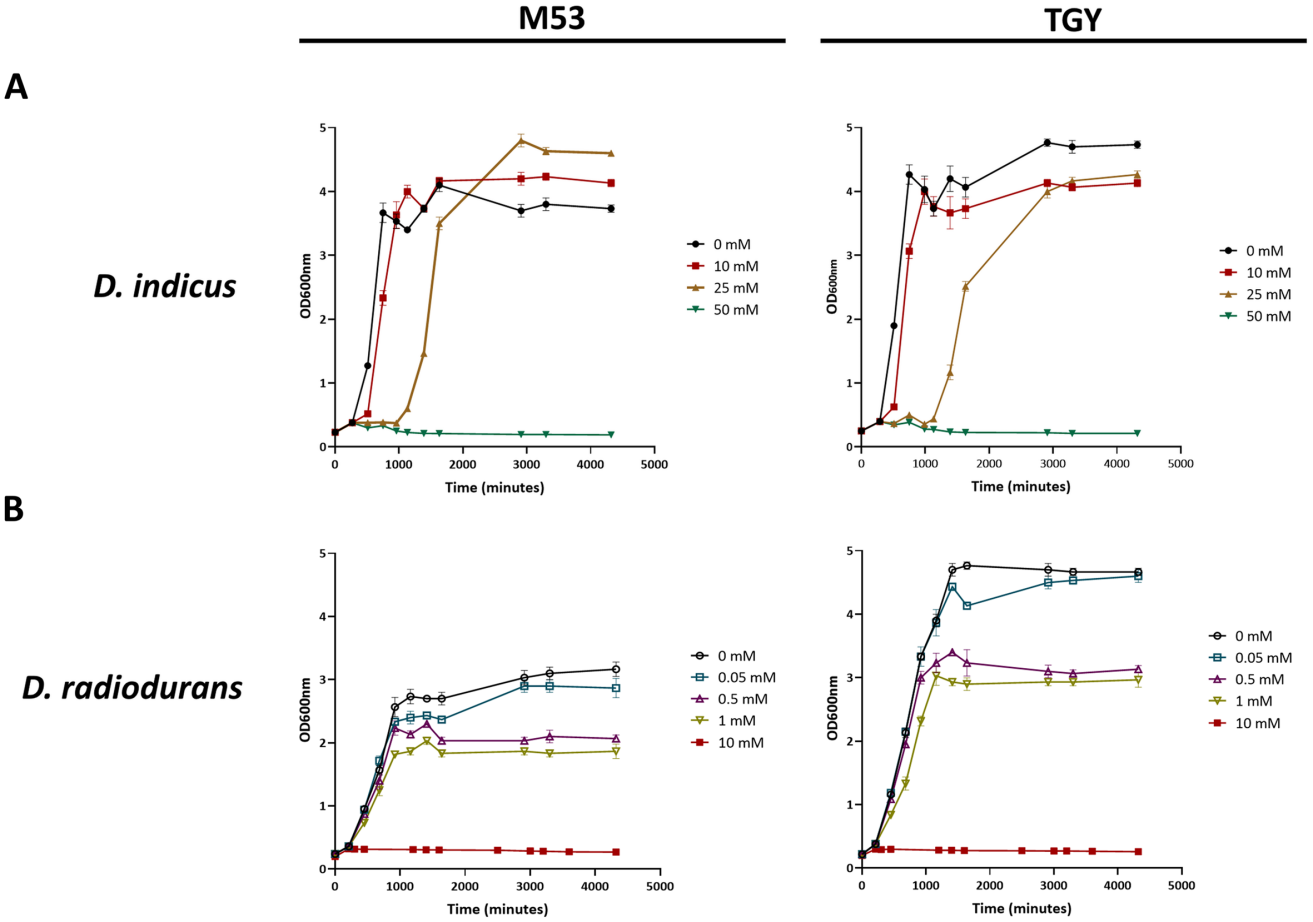
Growth curves in TGY and M53 media cultures of *Deinococcus* cells exposed to increasing concentrations of As(V) at an early exponential phase (OD_600_ = 0.3). **(A)** *D. indicus*, filled circles correspond to 0 mM of As(V), filled squares correspond to 10 mM of As(V), filled right side up triangles correspond to 25 mM of As(V) and filled upside down triangles correspond to 50 mM of As(V). **(B)** *D. radiodurans*, open circles correspond to 0 mM of As(V), open squares correspond to 0.05 mM of As(V), open right side up triangles correspond to 0.5 mM of As(V), open upside down triangles correspond to 1 mM of As(V) and filled squares correspond to 10 mM of As(V).

*Deinococcus indicus* Wt/1aT was originally isolated from an aquifer in West Bengal, India, and it was reported to survive 10 mM of As(V) in nutrient broth medium (Suresh et al., 2004). To evaluate its ability to cope with higher concentrations of As(V), *D. indicus* and *D. radiodurans* (non-arsenic resistant specie) were exposed to As(V) in their early exponential growth phase (OD_600_ = 0.3). *D. indicus* was able to grow in the presence of 25 mM As(V) in both media cultures. However, an extended lag phase was observed and at 50 mM As(V), cells were unable to grow (Figure 3A). Nevertheless, cells harvested and applied to fresh media were able to recover their growth in normal conditions (Supplementary Figure S1). Regarding *D. radiodurans*, different As(V) concentrations were tested, namely 0.05, 0.5, 1, 5, 10, and 20 mM, our results show that in the presence of 5, 10, or 20 mM, the cells are not able to recover the growth after 24H (Figure 3B). Cells were only able to display normal growth in 50 μM As(V), a 500-fold lower concentration than applied to *D. indicus*. When exposed to 1 mM, an evident decrease in growth was observed (Figure 3B). This observation was more evident in cells grown in TGY than for those grown in M53.

**Figure 4:**
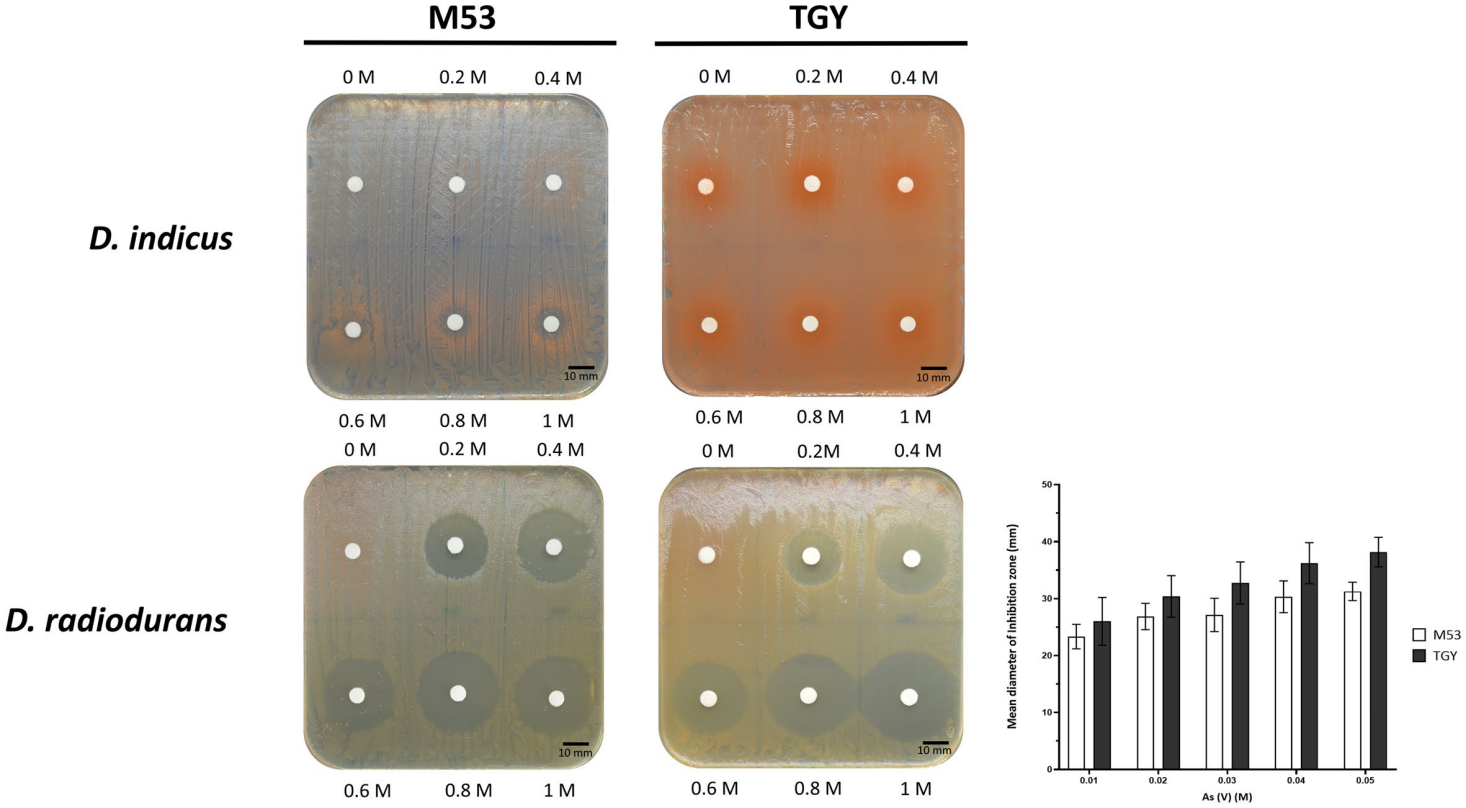
Survival of *D. indicus* and *D. radiodurans* in solid matrix (M53 and TGY culture media) in response to As(V). Cells at mid-exponential phase (OD_600_ = 2.0) were exposed to increasing concentrations of As(V) using an adapted Kirby-Bauer assay (Bauer et al., 1966; Hudzicki, 2012). Inhibition halos correspond to the mean diameter of inhibition zone (mm). The error bars represent the standard deviations from three replicates of four independent experiments (*n* = 4).

To further assess their resistance to As(V) in a solid matrix, *D. indicus* and *D. radiodurans* were exposed to increasing concentrations of arsenate ranging from 200 mM to 1 M (Figure 4). Interestingly, in the solid matrix *D. indicus* was not fully inhibited by 1 M of As(V) in either culture medium. Nevertheless, partial inhibition halos (reduction of cellular density) were observed in cells grown in M53 upon submission to 600 mM to 1 M of As(V). In contrast, *D. radiodurans* is inhibited by 200 mM of arsenate (Figure 4).

### SEM of *Deinococcus indicus*

**Figure 5:**
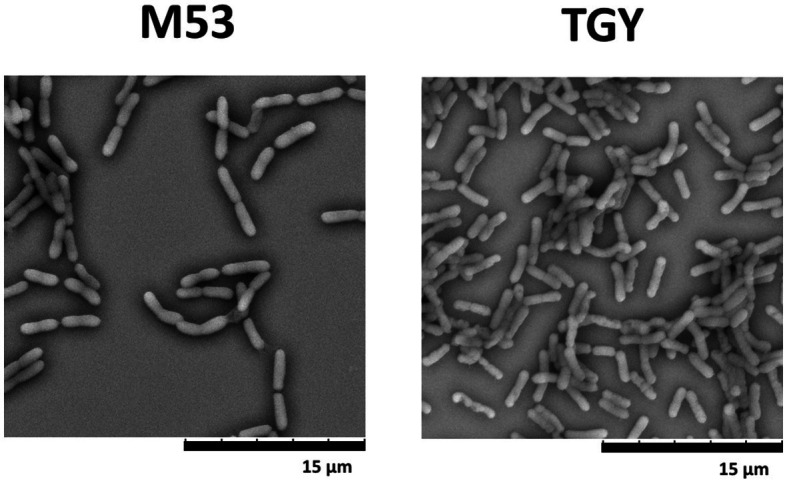
Scanning electron microscopy (SEM) images of *D. indicus* cells grown in M53 and TGY.

Previously, it was demonstrated that *D. indicus* presents growth media-induced morphotypes displaying a reversible behavior from single cells to multi-cell chain morphology (Chauhan et al., 2019b). To explore this capacity and understand if differences in morphotypes account for the behavior disparities observed against cell insults, *D. indicus* grown in M53 and TGY media were observed through Scanning Electron Microscopy (SEM). SEM data revealed small differences on the size of the cells, where *D. indicus* grown in TGY presented slightly lower cell lengths. However, no differences in cell chain clustering were observed (Figure 5). Nevertheless, the addition of 500 μM MnCl_2_ seems to affect cells grown in TGY, where cell septation is compromised and cell length is vastly increased (Supplementary Figure S2).

### Cryo-STEM EDX of *Deinococcus indicus*

**Figure 6:**
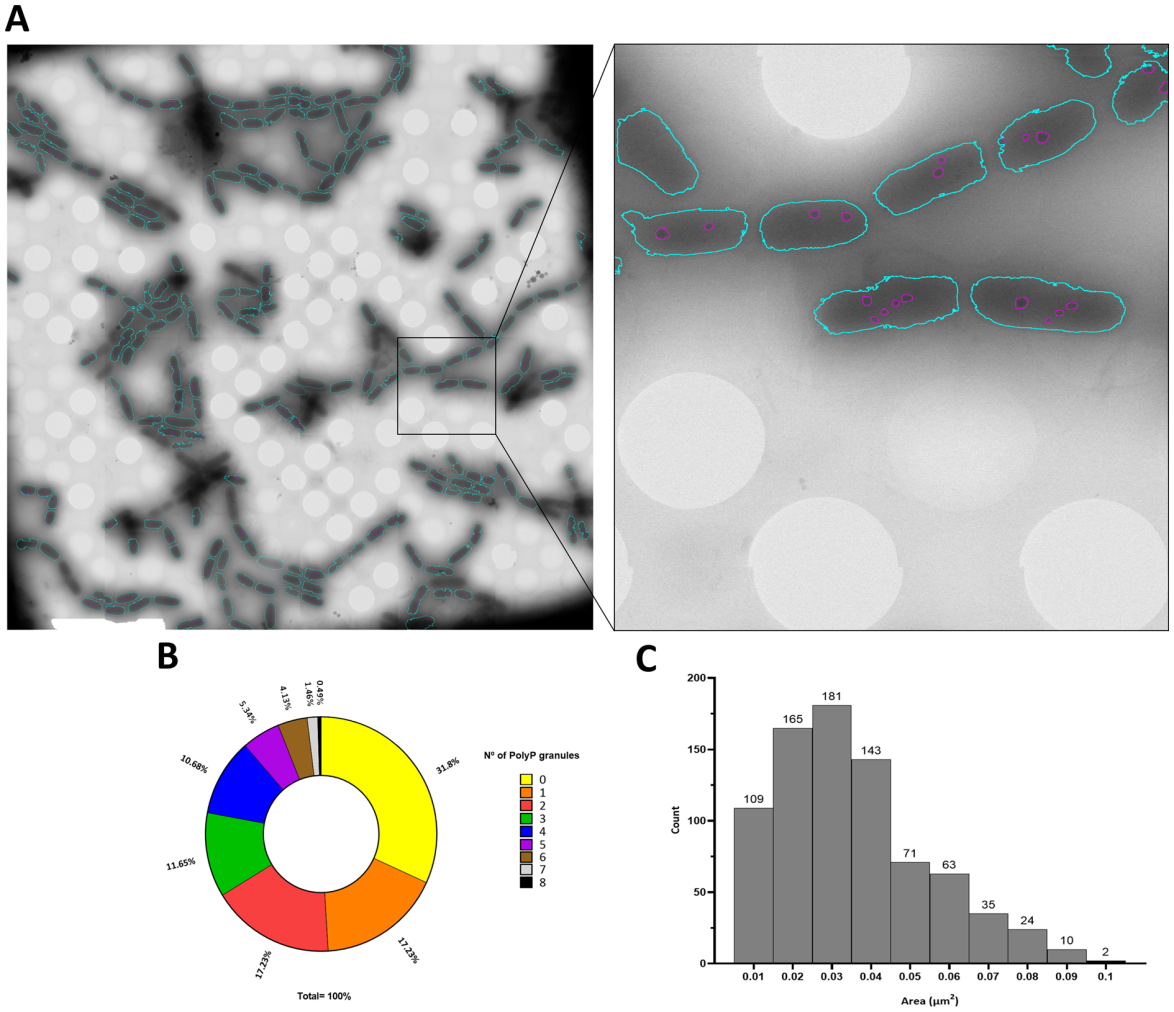
PolyP granules analysis by Cryo-STEM. **(A)** *D. indicus* STEM stitched image segmented trough the trained classifier, detected bacteria are colored in blue and granules within the bacteria are colored in purple. **(B)** Percentage of cells containing PolyP granules. **(C)** Area (μm^2^) of *D. indicus* PolyP granules.

**Figure 7:**
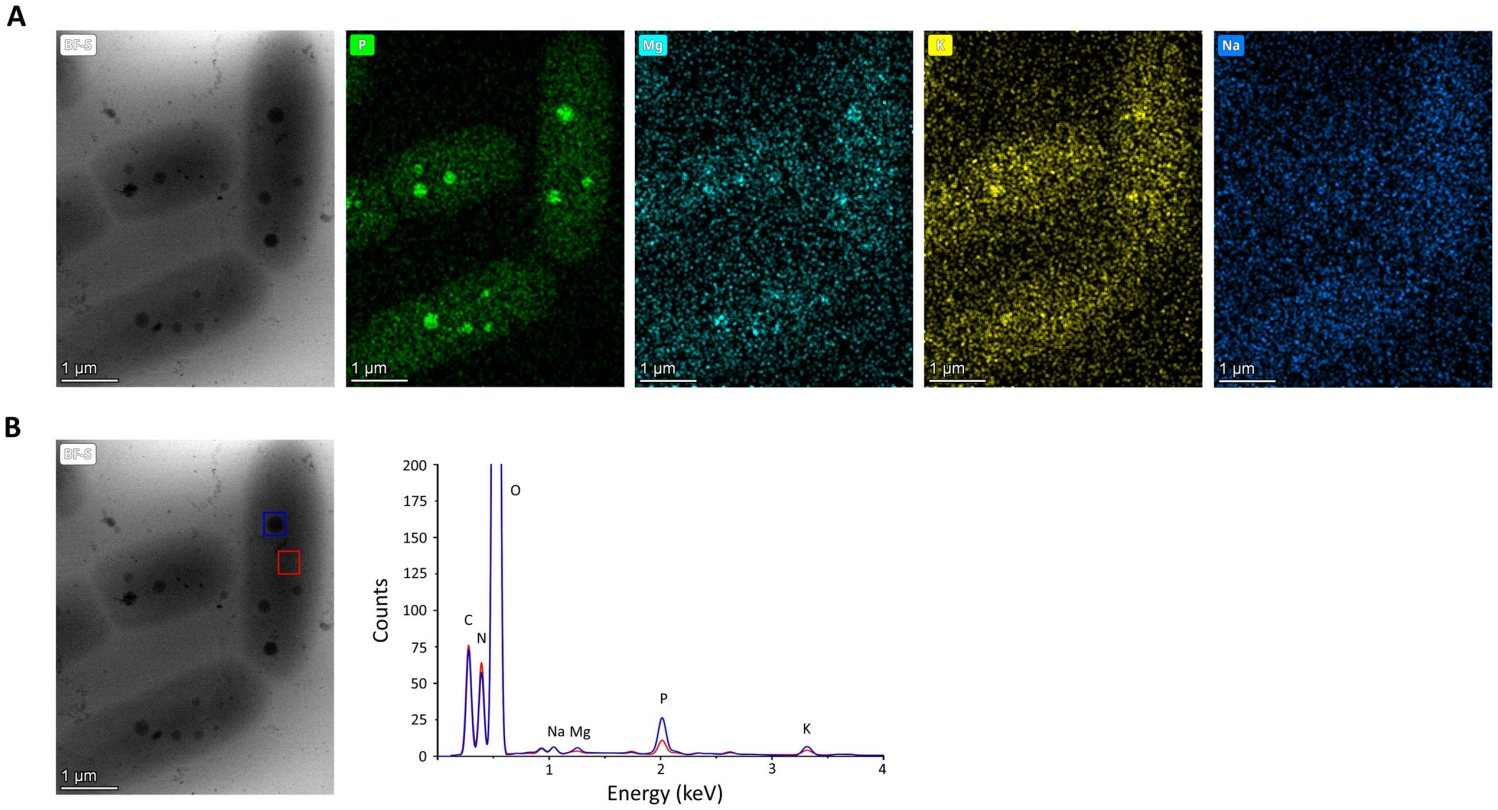
EDX analysis of *D. indicus* cells grown in M53. **(A)** Cryo-STEM images of control condition for which EDX spectra were measured, and elemental mapping for phosphorus (green), magnesium (cyan), potassium (yellow), and sodium (dark blue). **(B)** Blue square highlights a polyphosphate granule and red square a cytosol region, used to obtain the superimposed EDX spectra the polyphosphate granule (blue line) and the cytosol (red line).

Polyphosphate-like granules (pPLGs) are known to be involved in the sequestration of cation ions as well as in heavy-metal detoxification (Peverly et al., 1978; Urech et al., 1978; Scott and Palmer, 1990; Keasling, 1997; Kulakovskaya, 2018). *D. radiodurans, Deinococcus murrayi* and *Deinococcus proteolyticus* present pPLGs in their native compositions, but no information is available for *D. indicus* (Thornley et al., 1965; Sleytr et al., 1976; Ferreira et al., 1997; Eltsov and Dubochet, 2005; Copeland et al., 2012). Wolf et al. (2015) was carried out to image *D. indicus* in native conditions at the early-exponential phase in M53 medium. Through the ImageJ macro previously trained using ilastik “autocontext” approach, we were able to rapidly quantify and segment *D. indicus* cells and their granules using Cryo-STEM stitched images (Figure 6A). We observed a wide heterogeneity of electron dense granules distribution ranging from 0 to 8 granules per cell (Figure 6B). Overall, from the 435 cells analyzed 31.8% of the cells had no granules, followed by 17.2% of the cells containing 1 or 2 granules. Only 1.4 and 0.5% of the cells contained 7 and 8 granules, respectively (Figure 6B). The average granule area (*n* = 803) was 0.040 ± 0.019 μm^2^ with the smallest granules measuring 0.012 μm^2^ while the biggest granule measuring 0.107 μm^2^ (Figure 6C). EDX spectral analysis detected the enrichment in phosphorus of the intracellular granules, confirming that these structures are indeed polyphosphate-like granules (pPLGs) (Figure 7). The main counter ion found was magnesium (Supplementary Table S3).

### *Di*ArsC2 (arsenate reductase) phylogenetic analysis

**Figure 8:**
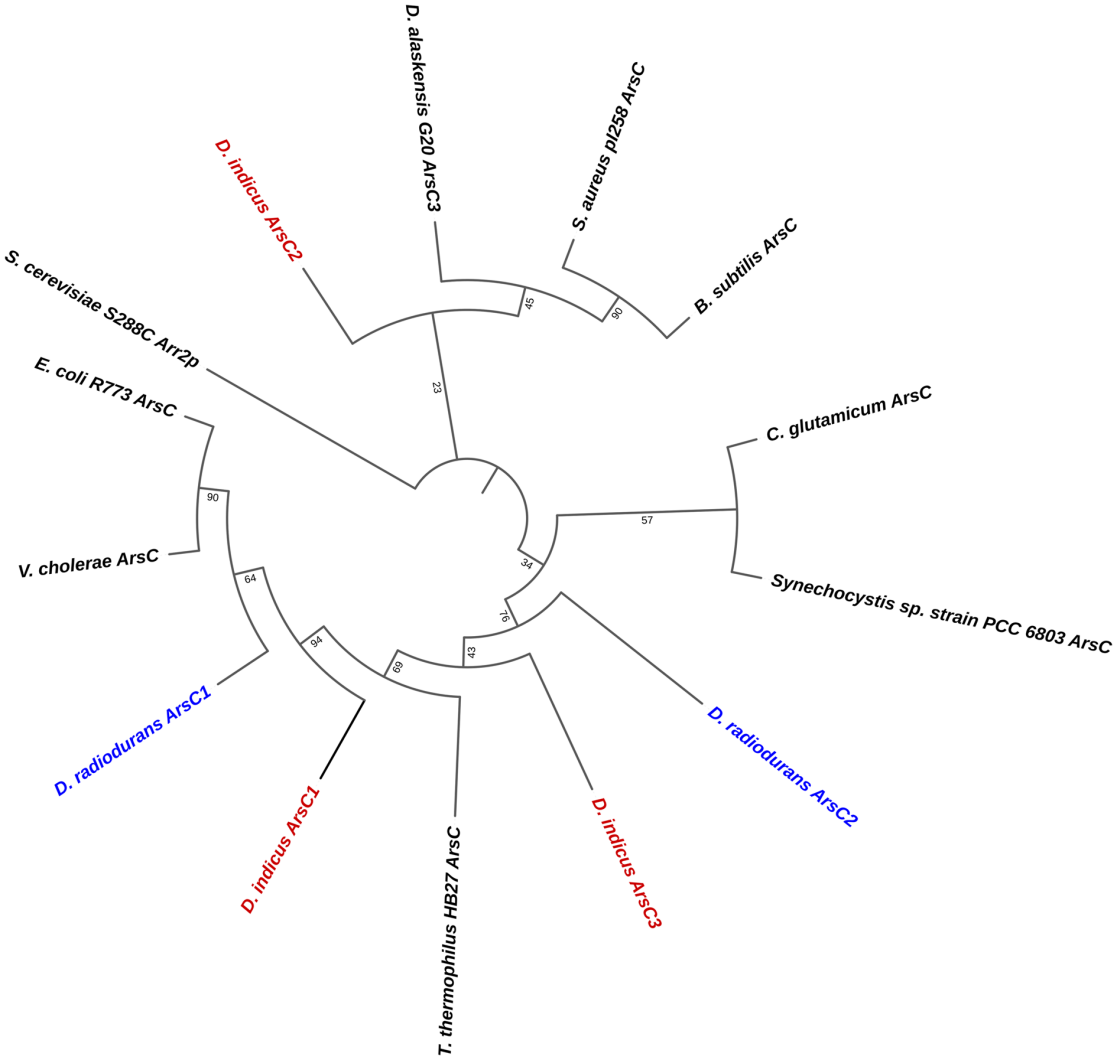
Phylogenetic tree of the three families of cytosolic arsenate reductases. Accession numbers for the amino acid sequences are: *B. subtilis* (P45947), *Corynebacterium glutamicum* (Q8NQC6), *D. indicus* ArsC1 (GHG23001.1), *D. indicus* ArsC2 (WP_088246862.1), *D. indicus* ArsC3 (WP_191300003.1), *D. radiodurans* ArsC1 (UDK99340.1), *D. radiodurans* ArsC2 (ANC72982.1), *Desulfovibrio alaskensis G20* (WP_011368603.1), *E. coli* R773 (P08692.1), *Saccharomyces cerevisiae* S288C Arr2P (DAA11614.1) *S. aureus* pl258 (P0A006), *Synechocystis* sp. PCC 6803 (P74313),*Thermus thermophilus* HB27 (AAS81844.1) and *Vibrio cholerae* (Q9KQ39). The three *D. indicus* ArsC and two *D. radiodurans* ArsC are shown in red and blue, respectively.

In order to explore the difference between the two species, *D. radiodurans* and *D. indicus*, to the arsenate resistance we analyzed the arsenate reductases that belong to the *ars* operon, which are able to convert As(V) into As(III) (Mukhopadhyay and Rosen, 2002). It is predicted that *D. indicus* possesses three genes that encode cytosolic arsenate reductases, *Di*ArsC1, *Di*ArsC2, and *Di*ArsC3 while *D. radiodurans* possesses two arsenate reductases, *Dr*ArsC1 and *Dr*ArsC2 (Figure 8). There are three families of cytosolic arsenate reductases: one belongs to the *E. coli* R773 plasmid and comprises glutaredoxin (Grx)-coupled enzymes, while another class belongs to the *Staphylococcus aureus* Plasmid pI258 comprising the thioredoxin (Trx)-coupled enzymes (Ji et al., 1994; Oden et al., 1994; Shi et al., 1999; Messens et al., 2002a; Messens and Silver, 2006) and the final class belongs to eukaryotic organisms, encompassing the ACR2p from *Saccharomyces cerevisiae* (Mukhopadhyay and Rosen, 1998; Mukhopadhyay et al., 2000). A sequence analysis of the different protein sequence, shows that the three arsenate reductases from *D. indicus, Di*ArsC1, *Di*ArsC2 and *Di*ArsC3 belong to three branches (Figure 8). Interestingly, *Di*ArsC1 and *Dr*ArsC1 branch closely to the *E. coli* ArsC from the Grx-linked prokaryotic family (Figure 8; Gladysheva et al., 1994; Rosen, 1999). *Di*ArsC3 and *Dr*ArsC2 branch closely to *Thermus thermophilus* ArsC that belongs to the Trx-linked prokaryotic ArsC reductases family. *Di*ArsC2 branch closely to *Desulfovibrio alaskensis* G20 ArsC3 (Nunes et al., 2014), which are in the main branch with *S. aureus* pI258 ArsC (Ji et al., 1994; Rosen, 1999; Figure 8). Since *Di*ArsC2 did not cluster together with any of *D. radiodurans* ArsCs, we decided to further unveil the structural mechanism for arsenate detoxification.

### *Di*ArsC2 and *Di*ArsC2-As structural insights

**Figure 9:**
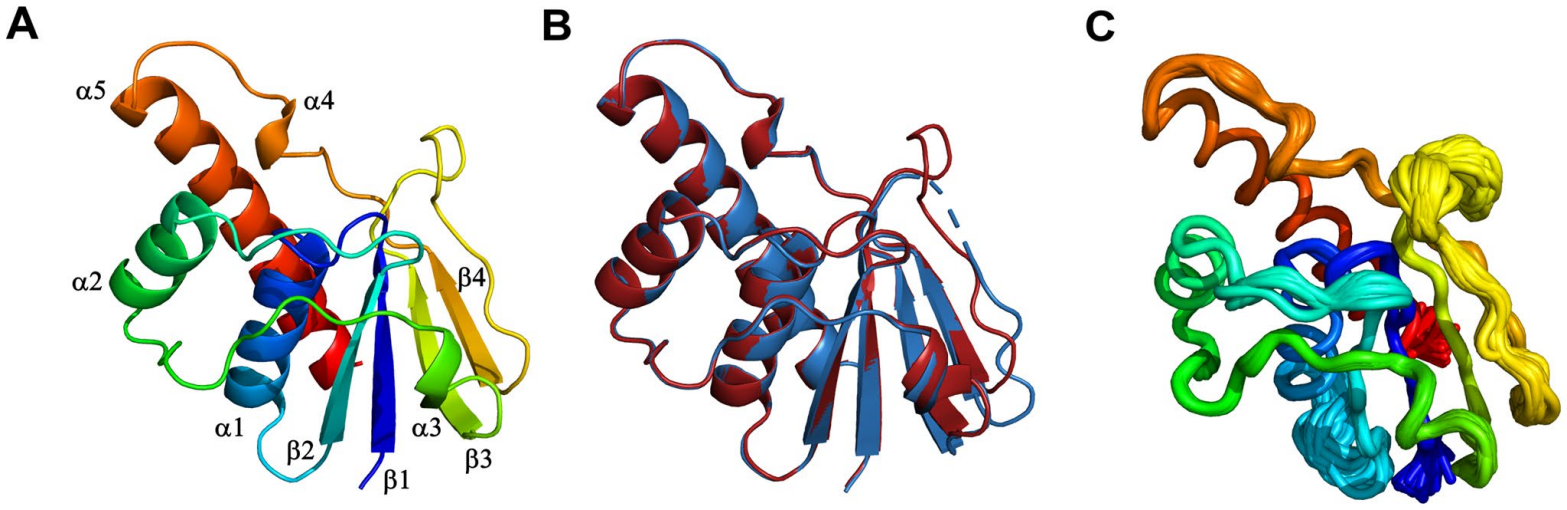
X-ray crystal structure of *D. indicus* arsenate reductase ArsC2. **(A)** Cartoon representation of the *Di*ArsC2-As, rainbow colored from blue (N-terminal) to red (C-terminal). **(B)** Superposition of the *Di*ArsC2-As (red) with the *Di*ArsC2 (blue) monomers. **(C)** C^α^ tube representation of the *Di*ArsC2-As monomer from the Ensemble refinement calculations.

**Figure 10:**
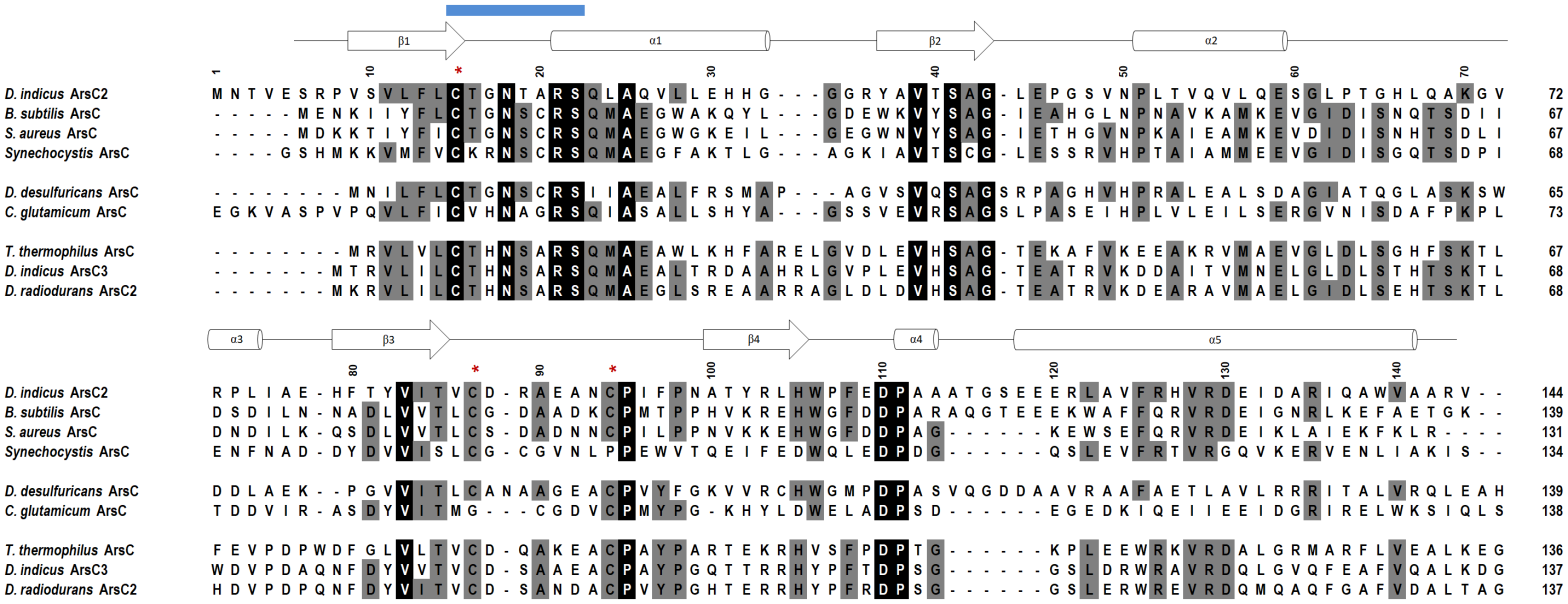
Amino acid sequence alignment of the *D. indicus* arsenate reductase ArsC2 with the Trx-linked family ArsC. The percentage identity of the ArsC of Trx-linked family with *D. indicus* ArsC2 (WP_088246862.1) is showed below. *B. subtilis* (34%, P45947); *S. aureus* pl258 (29%, P0A006); *Synechocystis* sp. PCC 6803 (27%, P74313); *D. desulfuricans* IC1 (26%, QCC86315.1); *C. glutamicum* (19%, Q8NQC6); *T. thermophilus* HB27 (28%, AAS81844.1); *D. indicus* ArsC3 (30%, WP_191300003.1); *D. radiodurans* ArsC2 (31%, ANC72982.1). *Di*ArsC2 secondary structure is shown above the alignment, and is indicated as α-helices and β-chains based on the output from PROCHECK (Laskowski et al., 1993). The three cysteine residues are marked with a red *. Amino acid residues that are part of the phosphate-binding loop are marked with a blue line. Strictly conserved amino acids represented as black boxes, whereas gray boxes represent the mostly conserved residues among the selected sequences.

The crystal structures of *Di*ArsC2 and *Di*ArsC2-As were determined and refined at resolutions of 1.65 and 1.50 Å, respectively. The space group of both crystal structures is *P*2_1_, presenting two molecules in the asymmetric unit. *Di*ArsC2 and *Di*ArsC2-As structures were refined to final *R*_factor_/*R*_free_ values of 0.224/0.254 and 0.152/0.172, respectively (Supplementary Table S2). The superposition of both chains based on the secondary structure matching algorithm yields a root-mean-square deviation (r.m.s.d.) of 0.6 Å between the superimposed C^α^ carbon atoms and 127 aligned amino acid residues. The largest deviations are in a loop region between residues 88–100 that presents high flexibility and for *Di*ArsC2 it lacks electron density for this region (Figures 9A,B). In fact, in *Di*ArsC2 it was not possible to build the protein chain between the residues 89–96 for Chain A and 89–93 for Chain B (Figure 9B). Thus, the structural analysis for both structures were performed with Chain B. Ensemble refinements (Burnley et al., 2012) were performed for *Di*ArsC2 and *Di*ArsC2-As (leading to *R*_factor_/*R*_free_ values of 0.221/0.255 and 0.160/0.177 respectively) where the most flexible regions were residues 31–36 and the loop 86–97 that contains the two conserved cysteines (Cys87 and Cys94) involved in As(V) reduction (Figures 9C, 10). The loop that contains the conserved catalytic Cys15 does not show a high degree of structural flexibility between the two structures here presented.

**Figure 11:**
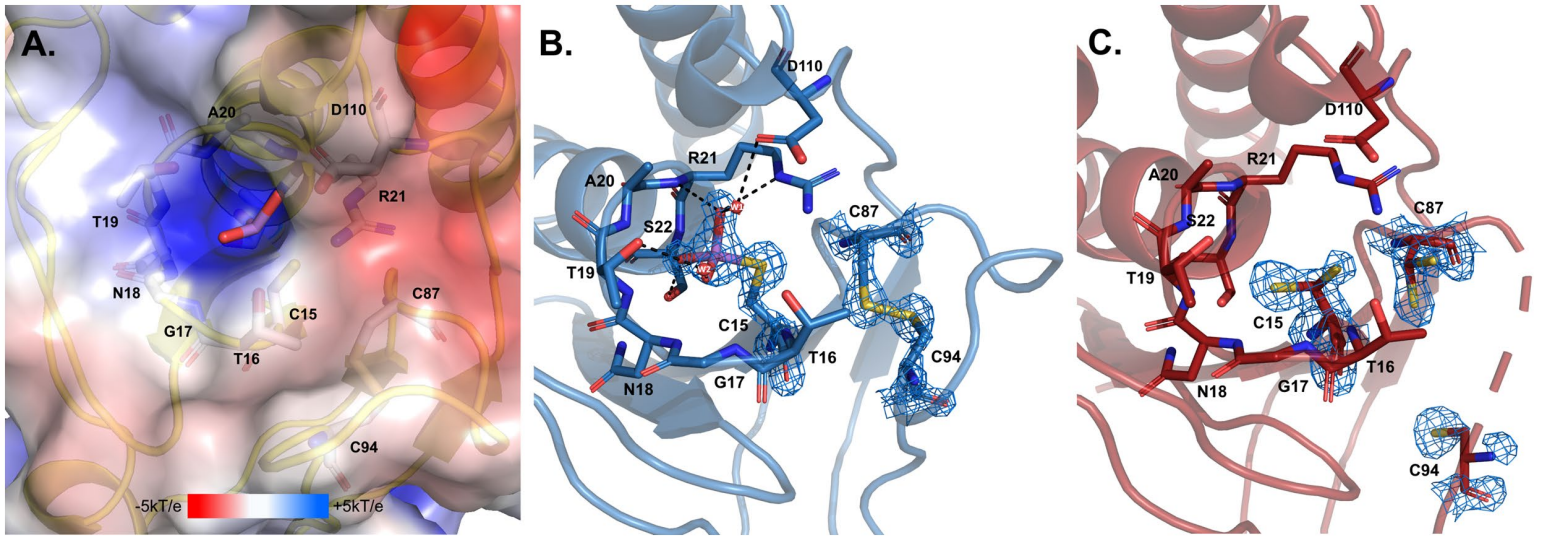
Arsenate catalytic site of *D. indicus* ArsC2. **(A)** Electrostatic potential mapped on the molecular surface of the pocket where arsenate enters, calculated by APBS (Baker et al., 2001; Dolinsky et al., 2004) integrated in the PYMOL (Delano, 2022). **(B)** *Di*ArsC2–As and **(C)** *Di*ArsC2 catalytic centers. In all the panels structures are shown in cartoon representation, with selected amino acid residues and arsenate drawn as sticks. In **(B,C)**, the final σ_A_-weighted 2|F_o_|–|F_c_| electron density map is represented at the 2 σ and 1 σ contour levels, respectively around the cysteine residues and the arsenate ligand. In **(A–C)** carbons are colored in gray, blue and red, respectively; nitrogen in dark blue, oxygen in red, sulfur in yellow, and arsenic in purple. Two water molecules W1 and W2 are represented as small red spheres. Black dashes represent the hydrogen bond interaction with neighboring atoms to the arsenate and the water molecules.

*Di*ArsC2 structures are composed of an α/β domain and four parallel β-strands which are flanked by three helices and two small helices (Figures 10, 11). These crystal structures are similar to the arsenate reductases from *B. subtilis* (Bennett et al., 2001), (PDB 1JL3) and *S. aureus*, (PDB 1LJL) (Messens et al., 2002b). The crystal structures of *Di*ArsC2 and *Di*ArsC2-As presented an r.m.s.d. value of 1.3 Å with 127 aligned amino acid residues with the arsenate reductases from *B. subtilis* (Bennett et al., 2001) (PDB 1JL3). The arsenate reductase from *S. aureus*, (PDB 1LJL) presents an r.m.s.d. value of 1.0 Å and 119 aligned amino acid residues with *Di*ArsC2 and an r.m.s.d. value of 1.2 Å and 121 aligned amino acid residues with *Di*ArsC2-As.

The reduction mechanism of arsenate involves three cysteines, Cys15, Cys87, and Cys94. In the case of the *Di*ArsC2-As structure, a disulfide bridge between Cys87 and Cys94 is formed. Although the protein was co-crystallized in the presence of arsenate (AsO_4_^3−^), the density observed close to the catalytic Cys15 suggests the presence of AsHO_3_^2−^. This molecule is located in a positively charged pocket and establishes a network of hydrogen bonds with the main-chain nitrogen atoms of residues G_17_NTARS_22_, Thr16 and Ser22 hydroxyl and Arg21^Nε^ groups, as well as water molecules (Figures 11A,B). Cysteine 15 is part of a well-known phosphate-binding loop (motif, CX_5_R) of LMW PTPases which is conserved in the Trx-linked ArsC family (Figure 10; Bennett et al., 2001). In the native structure, there is no species present in this pocket, and all the residues in the phosphate binding loop G_17_NTARS_22_, are in a similar position as in the Arsenate-bound structure. Although no electron density was observed for the region 89–93 (Chain B), weak density for Cys94 was obtained (Figure 11C). Cys15 and Cys87 are involved in the reduction of As(V) to As(III), and in *Di*ArsC2 structure both residues were refined with a double side-chain conformation, suggesting the presence of a mixed state (Figure 11C).

## Discussion

*Deinococcus* genus is ubiquitously present in nature and is able to tolerate high levels of radiation insults (Jin et al., 2019). *D. indicus* is a Gram-negative and arsenic resistant bacterium that presents a rod-shaped morphology in contrast to most *Deinococcus* sp. including *D. radiodurans* that present a coccus morphology (Suresh et al., 2004). It is known that arsenic induces oxidative stress in both eukaryotic and prokaryotic organisms (Huang et al., 2010; Ciprandi et al., 2012; Belfiore et al., 2013; Andres and Bertin, 2016; Shah and Damare, 2018; Castro-Severyn et al., 2019; Hu et al., 2020; De Francisco et al., 2021). Here, we report an extensive characterization of *D. indicus* resistance to oxidative stress, UV-C and arsenate. Oxidative stress analysis revealed that different culture conditions promote dissimilar responses to oxidative stress agents, and *D. indicus* presents a higher resistance to MV when grown in M53 than in TGY, while the same resistance pattern is observed in both media when exposing the cells to H_2_O_2_. Interestingly, *D. indicus* cells are more resistant to H_2_O_2_ than to MV. In bacteria, an increase in the expression of superoxide dismutase (SOD) and catalases has been observed upon exposure to arsenic (Weiss et al., 2009; Ciprandi et al., 2012; Belfiore et al., 2013; Andres and Bertin, 2016; Shah and Damare, 2018). It is predicted that *D. indicus* has two superoxide dismutases (SOD), a MnSOD (accession number: GHG29236.1) a Cu/Zn SOD (accession number GHG15276.1) and one catalase (accession number: GHG14730.1). Moreover, both the superoxide anion and H_2_O_2_ can damage proteins containing iron sulfur clusters with the release of Fe^2+^ that can react with hydrogen peroxide, leading to the formation of hydroxyl radicals by the Fenton reaction (Koppenol, 1993; Valentine et al., 1998; Kehrer, 2000; Kohen and Nyska, 2002). *D. indicus* also possesses one DNA-binding protein under starved conditions, Dps (accession number: GHG25447.1). *D. radiodurans* Dps proteins are known to sequester iron and manganese (Ishikawa et al., 2003; Santos et al., 2015). *D. indicus* Dps presents high identity (75.5%) with *Dr*Dps1 which is known to utilize hydrogen peroxide for the oxidation of Fe^2+^ to Fe^3+^(Santos et al., 2015). In addition, it has been previously reported that *D. radiodurans* protein extracts present a higher ROS scavenging ability than in *E. coli*, namely 30 fold-higher for H_2_O_2_ and 6 fold higher for O_2_^.-^ (Tian et al., 2004).

So far, there is no information regarding UV-C resistance of *D. indicus.* In fact, *D. indicus* was screened for UV-B when first isolated by Suresh and co-workers, presenting 2–4% survival at 50000 J/m^2^ in nutrient broth media (Suresh et al., 2004). Here, we showed that UV-C resistance in *D. indicus* is media-dependent: cells grown in M53 require a dose of 809 ± 66 J/m^2^ to eliminate 90% of cells, while cells grown in TGY required a lower dose of 619 ± 82 J/m^2^.

Moreover, *D. indicus* cells grown in M53 were also more resistant than the ones in TGY when subjected to MV. Interestingly, it has been previously reported that *D. radiodurans* response to UV-C exposure is also media-dependent (Chen et al., 2023). We can postulate that this media dependent response to MV and UV-C could indicate similar resistance mechanisms are in place and they act in a media dependent way, however the molecular mechanisms involved need to be further investigated.

Previously, it was demonstrated that *D. indicus* Wt/1aT can survive to 10 mM of arsenate As(V) (Suresh et al., 2004). To further extend arsenic resistance characterization, herein we showed that *D. indicus* is able to reach control conditions in the presence of 25 mM of As(V). Interestingly, in M53 medium and in the presence of 10 mM and 25 mM of arsenate, cells presented higher OD_600_ compared to control conditions, while the same behavior was not observed in TGY. This could be due to higher resistance to oxidative stress conditions, or perhaps other transcripts are modulated when cells are grown in M53. Moreover, an extended lag phase in the presence of 25 mM of arsenate before resuming normal growth was observed in both media although it was more pronounce when the cells were grown in TGY. This is a fairly common effect, for instance, methyl-mercury acetate causes an extended lag-phase on *Rhodopseudomonas capsulata* before resumption of normal growth (Nielsen and Sojka, 1979; Brochiero et al., 1984; Jaiganesh et al., 2012; Aljerf and AlMasri, 2018). In *E. coli*, cadmium quantum dots caused a longer lag-phase until normal proliferation was restored (Jaiganesh et al., 2012). Moreover, *D. indicus* growth was suppressed by the presence of 50 mM of As(V). However, upon As(V) removal, *D. indicus* was able to restore normal growth conditions. In this case, an extended lag-phase similar to post-antibiotic effect (PAE) noted on antibiotic applications was also observed (Zhanel et al., 1991; Li et al., 2016; Srimani et al., 2017). PAE is characterized as the time period after total removal of antibiotic during which no growth is observed. Our data for arsenic resistance is consistent with that for *D. indicus* strain DR1 isolated from the wetlands of Dadri, Uttar Pradesh, India (Chauhan et al., 2017, 2019a). Regarding *D. radiodurans,* no lag phase was observed upon exposure to 0.5 and 1 mM of As(V), even though cell growth was compromised. No differences were observed between media cultures in *D. radiodurans* and the same pattern was verified by exposing the cells to As(V) in a solid matrix. In contrast, *D. indicus* is able to survive up to 1 M of As(V) in solid matrix.

Since *D. indicus* presents higher resistance to MV, UV-C and a faster response and growth in M53 when submitted to As(V), a STEM-EDX analysis was performed. Here we reported for the first time the presence of polyphosphate-like granules (pPLGs) in *D. indicus.* Moreover, more than 30% of the cells contained no granules while a few cells contained up to 8 granules. This high heterogeneity reveals high plasticity in PolyP granules of *D. indicus* cells, and could indicate that cells adopt specific roles in the community, specializing in PolyP storage to gain an advantage upon environmental changes. pPLGs are also known to be involved in heavy-metal detoxification (Pan-Hou et al., 2001, 2002; Alvarez and Jerez, 2004; Ruiz et al., 2011; Orell et al., 2012; Jasso-Chávez et al., 2019; Sanz-Luque et al., 2020). In *Anabaena cylindrica* a higher accumulation of aluminum was observed on cells exhibiting pPLGs that were grown in a phosphate-rich medium (Annette et al., 1985). It is known that polyphosphate kinases (PPKs) are involved in the formation of inorganic polyphosphate (polyP) and pPLGs (Kornberg, 1999). In *E. coli,* higher resistance to Hg^2+^ was observed after the introduction of *ppk* into the *merA*-deleted *mer* operon (Pan-Hou et al., 2001, 2002). Moreover, it was proposed that polyP was involved in conversion of Hg^2+^ to a less toxic molecule via chelation mechanisms (Pan-Hou et al., 2001, 2002). Nevertheless, further analysis of *D. indicus* ability to utilize polyP for arsenic detoxification is required, especially since *D. radiodurans* also presents pPLGs and is sensitive to arsenic. It would be vital to understand if *D. indicus* pPLGs aid on the detoxification of arsenic either being directly involved or indirectly.

In order to complement our studies and to further understand the resistance to arsenate by *D. indicus* the arsenate reductase *DiArsC2* was chosen since it did not cluster with any of *ArsC* from *D. radiodurans.* Thus, we undertook the structural studies of *Di*ArsC2 and we were able to crystallize two different forms of the enzyme: native (*Di*ArsC2) and bound to arsenite (*Di*ArsC2-As). The crystal structures present high similarities with the *B. subtillis* ArsC that belongs to the Trx-linked family (Bennett et al., 2001). Based on the conservation of the 3 cysteines (Cys15, Cys87, and Cys94) (Figure 10) and structural analysis (Figures 9, 11), we infer the As(V) mechanism of reduction is identical to the one proposed for *B. subtillis,* i.e., a triple redox system (Bennett et al., 2001). The *Di*Arsc2-As structure represents the final stage, where Cys15 is bound to As(III), however the ligand cannot be released since Cys87 forms a disulfide bridge with Cys94 (Figure 11). Moreover, the arsenate is located in a positive-charged pocket which is accessible from the external environment. Since in our *Di*ArsC2 structure the disulfide bridge between these two residues is not formed (at least in chain B, where density is observed) this could indicate that an As(V) reduction event already took place in the *Di*ArsC2-As structure, leading to the formation of the Cys87 – Cys94 disulfide bridge (Figure 11). The lack of mobility of the PTPase loop (where Cys15 is located) together with the high flexibility of the loop that contains Cys87 and Cys94 suggests that Cys87 is the residue responsible for the release of As(III) bound to the Cys15 (Figure 9). In fact this loop is readily accessible by the thioredoxin reductase system to reduce the Cys87 – Cys94 disulfide bridge and allow a new As(V) reduction cycle to occur (Messens et al., 1999; Bennett et al., 2001). It would be interesting to assess whether the presence of Cys87 in the *Di*ArsC2 structure is essential for the ligation of As(V). Moreover, the mutation of Cys87 could allow *Di*ArsC2 to be used has a tool for of As(V) removal by inhibiting the release of As(III).

## Conclusion

Arsenic contamination severely damages ecosystems and compromises the integrity of water resources essential for human life. *D. indicus* is an arsenic resistant organism belonging to Deinococcaceae family that present high resistant to UV radiation. Here we performed a characterization of stress resistance of *D. indicus* and provide insights toward exploiting *D. indicus* as a tool for bioremediation. *D. indicus* exhibited a media-dependent response upon exposure to UV-C and MV, and demonstrated higher resistance in M53 compared to TGY. Moreover, cells grown in M53 upon exposure to 25 mM As(V) exhibited higher growth (OD_600_). This may indicate that optimization of culture conditions could lead to a higher resistance to cellular insults including arsenic stress. Using STEM-EDX we were able to detect the presence of polyphosphate granules *in D. indicus*. Nevertheless, more studies are required to infer the potential utilization as a path for the detoxification process via arsenic bioaccumulation. Additionally, the *D. indicus ars* operon contains two arsenate reductases (*Di*ArsC2 and *Di*ArsC3). The *Di*ArsC2 structure revealed detailed insights into the molecular mechanism of the arsenate reduction, in which As(V) is converted to As(III) and remains bound to Cys15. These findings, contribute to the knowledge to apply *D. indicus* in bioremediation of arsenic either by following a cell-based approach or at the protein level using *Di*ArsC has a tool to remove As(V).

## Data availability statement

The original contributions presented in the study are included in the article/Supplementary material, further inquiries can be directed to the corresponding author.

## Author contributions

AG and CR: conceptualization and experimental design. AG, BS, WA, SW, ME, PM, and CR: methodology. CR, AG, SW, ME, and PK: STEM experiments. CR, AG, SW, OG, and DR: software, granules analysis, and data curation. WA and AG: SEM experiments. AG, BS, CR, and PM: protein purification and structure determination. AG and CR: UV-C, oxidative, and As(V) experiments. AG and CR analyzed the data and wrote the manuscript. CR: investigation, supervision, and project administration. AG, BS, DR, WA, PK, OG, SW, ME, PM, and CR: review and editing the manuscript. CR, SW, and ME: funding acquisition. All authors contributed to the article and approved the submitted version.

## Funding

This study was financially supported by the Portuguese Fundação para a Ciência e Tecnologia (FCT), grants PTDC/BIA-BQM/31317/2017, Project MOSTMICRO-ITQB with references UIDB/04612/2020 and UIDP/04612/2020, and LS4FUTURE Associated Laboratory (LA/P/0087/2020). This project has received funding from the European Union’s Horizon 2020 research and innovation program under grant agreement No. 857203. AG and BS are recipients of FCT grants SFRH/BD/06723/2020 and SFRH/BD/08066/2020, respectively. CR is recipient of FCT Institutional CEEC. Cryo-electron microscopy studies received partial support from the Weizmann Institute of Science (The Irving and Cherna Moskowitz Center for Nano and BioNano Imaging), and from the European Union (ERC-AdV grant, CryoSTEM, 101055413 to ME). This work benefited from access to the Weizmann Institute Electron Microscopy Unit, an Instruct-ERIC centre through the Access proposal PID: 19879.

## Conflict of interest

The authors declare that the research was conducted in the absence of any commercial or financial relationships that could be construed as a potential conflict of interest.

## Publisher’s note

All claims expressed in this article are solely those of the authors and do not necessarily represent those of their affiliated organizations, or those of the publisher, the editors and the reviewers. Any product that may be evaluated in this article, or claim that may be made by its manufacturer, is not guaranteed or endorsed by the publisher.
