# Supplementary material for: Unraveling the multifaceted resilience of arsenic resistant bacterium Deinococcus indicus
Source: Front Microbiol. 2023 Aug 24;14:1240798. doi: 10.3389/fmicb.2023.1240798 (PMC10483234; doi:10.3389/fmicb.2023.1240798)
Supplement: Supplementary file 1 [file Table_1.DOCX]

**Table S1.** Data collection and processing statistics.

| **Data Collection** | **DiArsC2** | **DiArsC2-As** |
| --- | --- | --- |
| Beamline | ALBA XALOC | ALBA XALOC |
| Detector | PILATUS | PILATUS |
| Wavelength (Ǻ) | 0.979264 Å | 0.979264 Å |
| Space Group | *P* 2(1) | *P* 2(1) |
| Unit cell parameters: |  |  |
| a, b, c (Ǻ) | 43.67 39.40 72.38 | 43.51 39.82 72.19 |
| β (°) | 97.383 | 97.075 |
| **Data Processing** | AutoPROC / STARANISO | AutoPROC / STARANISO |
| Resolution limits of ellipsoid fitted to resolution cut-off surface (Å) | 1.754, 1.592, 1.597 | 1.466, 1.471, 1.596 |
| Resolution, spherical limits (Å)^a^ | 19.69-1.65 (1.68-1.65) | 19.91-1.50 (1.55-1.50) |
| Nr. Observations ^a^ | 65553 (2521) | 108851 (5309) |
| Unique reflections ^a^ | 25923 (994) | 35509 (1775) |
| Multiplicity ^a^ | 2.5 (2.5) | 3.1 (3) |
| Completeness, spherical (%)^a^ | 87.3 (64.0) | 89.7 (41.3) |
| Completeness, ellipsoidal (%)^a^ | 89.7 (90.2) | 93.4 (61.4) |
| R-merge (%) ^b^ | 10.2 (75.6) | 4.6 (51.1) |
| R-p.i.m. (%) ^c^ | 7.7 (55.1) | 3.1 (34.7) |
| <I/σ (I)> | 8.4 (2.0) | 10.6 (1.5) |
| CC ^1/2^ | 0.993 (0.485) | 0.999 (0.725) |
| Wilson B (Å^2^) | 29.476 | 25.657 |
| Z ^d^ | 2 | 2 |
| Estimated V_M_ ^e^ | 1.96 | 1.97 |
| Estimated Solvent Content (%) ^e^ | 37.26 | 37.57 |

^a^ Values in parentheses refer to the highest resolution shell; ^b^ R-merge = merging R-factor, (Σ_hkl_ Σ_i_ |*I*_i_(hkl) - <*I*(hkl)>|) / (Σ_hkl_ Σ_i_ *I*(hkl)) × 100 %; ^c^ R-p.i.m. = precision-independent R-factor, Σ_hkl_ [1/(N-1)]^1/2^ Σ_i_ |*I*_i_(hkl) - <*I*(hkl) >| / (Σ_hkl_ Σ_i_ *I*_i_(hkl)) × 100 % (Diederichs and Karplus, 1997). For each unique Bragg reflection with indices (hkl), *I*_i_ is the i-th observation of its intensity and N its multiplicity; ^d^ Nr. molecules in the asymmetric unit; ^e^ According to (Matthews, 1968).

|  |
| --- |

**References:**

Chen, V. B., Arendall, W. B., Headd, J. J., Keedy, D. A., Immormino, R. M., Kapral, G. J., et al. (2010). MolProbity: All-atom structure validation for macromolecular crystallography. *Acta Crystallogr. Sect. D Biol. Crystallogr.* 66, 12–21. doi: 10.1107/S0907444909042073.

Diederichs, K., and Karplus, P. A. (1997). Improved R-factors for diffraction data analysis in macromolecular crystallography. *Nat. Struct. Biol.* 4, 269–275. doi: 10.1038/nsb0497-269.

Liebschner, D., Afonine, P. V., Baker, M. L., Bunkoczi, G., Chen, V. B., Croll, T. I., et al. (2019). Macromolecular structure determination using X-rays, neutrons and electrons: Recent developments in Phenix. *Acta Crystallogr. Sect. D Struct. Biol.* 75, 861–877. doi: 10.1107/S2059798319011471.

Matthews, B. W. (1968). Solvent content of protein crystals. *J. Mol. Biol.* 33, 491–497. doi: 10.1016/0022-2836(68)90205-2.
