# Supplementary material for: Unraveling the multifaceted resilience of arsenic resistant bacterium Deinococcus indicus
Source: Front Microbiol. 2023 Aug 24;14:1240798. doi: 10.3389/fmicb.2023.1240798 (PMC10483234; doi:10.3389/fmicb.2023.1240798)
Supplement: Supplementary file 2 [file Table_2.DOCX]

**Table S2.** Refinement statistics.

| **Dataset** | ***Di*ArsC2** | ***Di*ArsC2-As** |
| --- | --- | --- |
| Resolution limits (Å)^a^ | 19.70-1.65 (19.70-1.65) | 19.91-1.50 (19.91-1.50) |
| *% R*_work_ ^b^ | 22.4 (25.4) | 15.2 (17.2) |
| *% R*_free_ ^c^ | 22.3 (25.3) | 15.2 (17.1) |
| ML coordinate error estimate (Å) ^d^ | 0.22 | 0.12 |
| *Model composition and completeness* |  |  |
| Regions omitted | Chain A: 1-7; 89-96  Chain B: 1-7; 89-93; 144 | Chain A:1-5; 144  Chain B: 1-7; 143-144 |
| Non-hydrogen protein atoms ^e^ | 2014 | 2149 |
| Ligand/ion | - | 2 |
| Solvent molecules | 262 | 307 |
| Glycerol molecules | - | 2 |
| *Mean B values (Å^2^)* ^f^ |  |  |
| Protein | Chain A: 23.2;  Chain B: 22.1 | Chain A: 19.7;  Chain B: 22.9 |
| Ligand/ion | - | 14.58 |
| Solvent | 28.19 | 30.64 |
| Glycerol | - | 44.13 |
| *Model r.m.s. deviations from ideality* |  |  |
| Bond lengths (Å) | 0.005 | 0.004 |
| Bond angles (°) | 0.674 | 0.670 |
| Chiral centers (Å^3^) | 0.044 | 0.059 |
| Planar groups (Å) | 0.005 | 0.006 |
| *Model validation* **^h^** |  |  |
| % Ramachandran outliers | 0 | 0 |
| % Ramachandran favored | 98.81 | 98.51 |
| % Rotamer outliers | 0 | 0 |
| % C^β^ outliers | 0 | 0 |
| Clash score | 2.76 | 0.94 |
| PDB Accession code | 8P6M | 8P5N |

^a^ Values in parentheses refer to the highest resolution shell; ^b^ R_work_ = (Σ_hkl_ ||F_obs_(hkl)| - |F_c8P5Malc_(hkl)||) / (Σ_hkl_ |F_obs_(hkl)|) × 100 %; ^c^ R_free_ is calculated as above from a random sample containing 5% of the total number of independent reflections measured; ^d^Maximum-likelihood estimate by PHENIX (Liebschner et al., 2019); ^e^ Including atoms in the alternate conformations of disordered groups of residues; **^g^** Calculated from isotropic or equivalent isotropic B-values; ^f^Calculated with MolProbity (Chen et al., 2010).

|  |
| --- |

**References:**

Chen, V. B., Arendall, W. B., Headd, J. J., Keedy, D. A., Immormino, R. M., Kapral, G. J., et al. (2010). MolProbity: All-atom structure validation for macromolecular crystallography. *Acta Crystallogr. Sect. D Biol. Crystallogr.* 66, 12–21. doi: 10.1107/S0907444909042073.

Diederichs, K., and Karplus, P. A. (1997). Improved R-factors for diffraction data analysis in macromolecular crystallography. *Nat. Struct. Biol.* 4, 269–275. doi: 10.1038/nsb0497-269.

Liebschner, D., Afonine, P. V., Baker, M. L., Bunkoczi, G., Chen, V. B., Croll, T. I., et al. (2019). Macromolecular structure determination using X-rays, neutrons and electrons: Recent developments in Phenix. *Acta Crystallogr. Sect. D Struct. Biol.* 75, 861–877. doi: 10.1107/S2059798319011471.

Matthews, B. W. (1968). Solvent content of protein crystals. *J. Mol. Biol.* 33, 491–497. doi: 10.1016/0022-2836(68)90205-2.
