# Supplementary material for: Unraveling the multifaceted resilience of arsenic resistant bacterium Deinococcus indicus
Source: Front Microbiol. 2023 Aug 24;14:1240798. doi: 10.3389/fmicb.2023.1240798 (PMC10483234; doi:10.3389/fmicb.2023.1240798)
Supplement: Supplementary file 3 [file Table_3.DOCX]

**Table S3.** STEM-EDX spectral analysis of *D. indicus* cells (n=5) pPLGs (n=16) and cytosol regions. The average atomic fraction of the measured elements based on the K-line family is given.

| Element | Atomic Fraction (%) | |
| --- | --- | --- |
|  | **pPLGs** | **Cytosol** |
| Na | 9,1 ± 2,9 | 23,8 ± 5,5 |
| Mg | 15,7 ± 3,3 | 14,2 ± 3,3 |
| P | 59,6 ± 4,6 | 36,8 ± 4,1 |
| Cl | 3,3 ± 1,1 | 8 ± 2,9 |
| K | 12,4 ± 3,3 | 17,7 ± 3,9 |
