# Supplementary figures and images for: Unraveling the multifaceted resilience of arsenic resistant bacterium Deinococcus indicus
Source: Front Microbiol. 2023 Aug 24;14:1240798. doi: 10.3389/fmicb.2023.1240798 (PMC10483234; doi:10.3389/fmicb.2023.1240798)

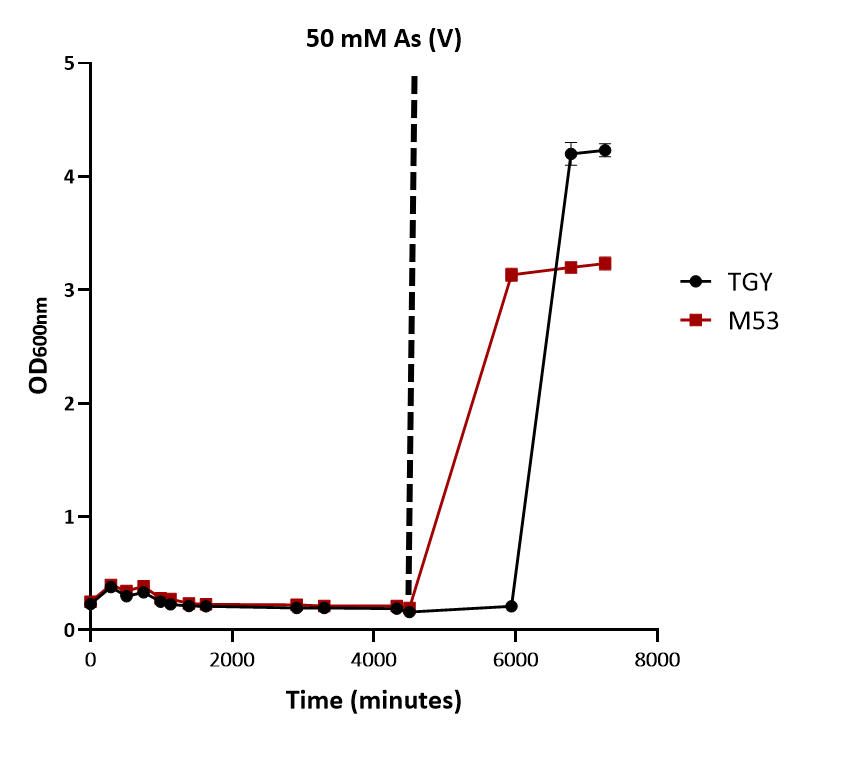

Supplement: Supplementary file 4 [file Image_1.TIFF]
